# Supplementary figures and images for: Epigenetic associations of type 2 diabetes and BMI in an Arab population
Source: Clin Epigenetics. 2016 Jan 28;8:13. doi: 10.1186/s13148-016-0177-6 (PMC4730771; doi:10.1186/s13148-016-0177-6)

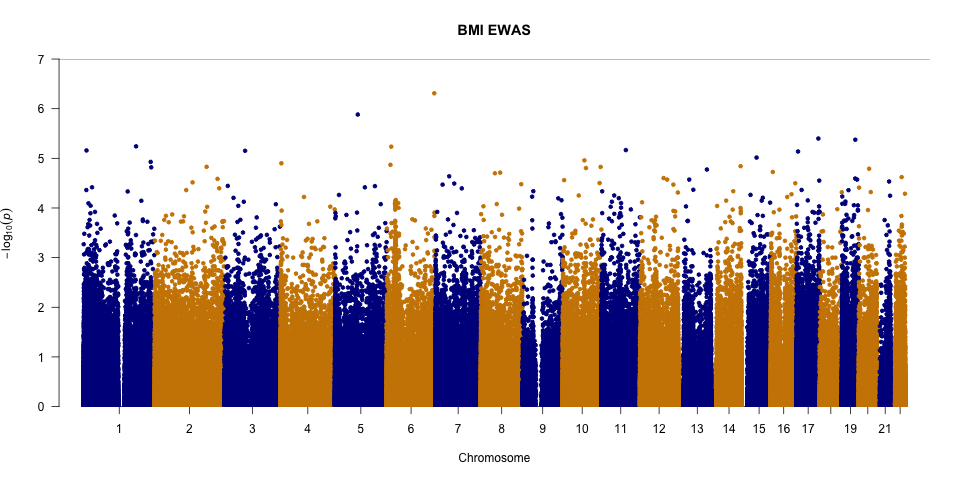

Supplement: Additional file 2: Figure S2. — Manhattan plots for epigenome-wide association of CpG methylation sites with (A) BMI (B) T2D. Coordinates are in hg19. The red line indicates a conservative Bonferroni significance threshold of p value = 1.07 × 10^−7^. 277 KB [file 13148_2016_177_MOESM2_ESM.zip › SupFig2a.png]

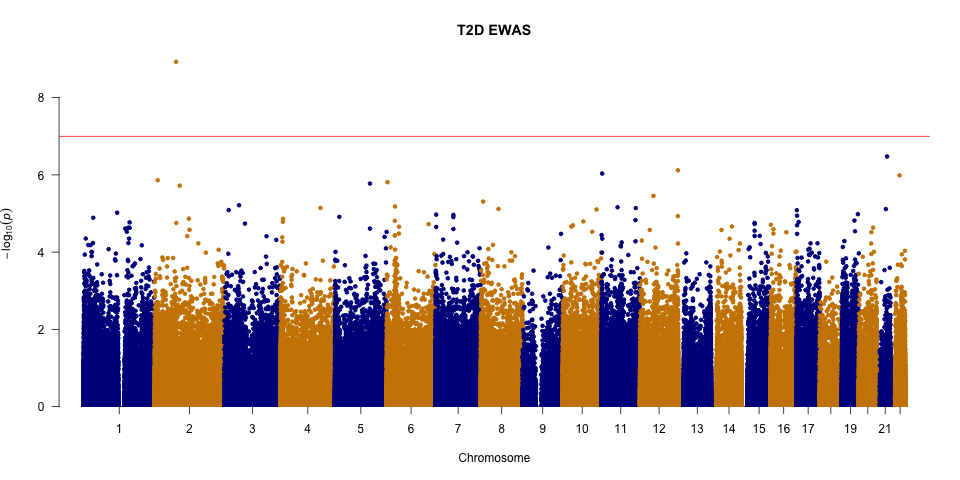

Supplement: Additional file 2: Figure S2. — Manhattan plots for epigenome-wide association of CpG methylation sites with (A) BMI (B) T2D. Coordinates are in hg19. The red line indicates a conservative Bonferroni significance threshold of p value = 1.07 × 10^−7^. 277 KB [file 13148_2016_177_MOESM2_ESM.zip › SupFig2b.png]

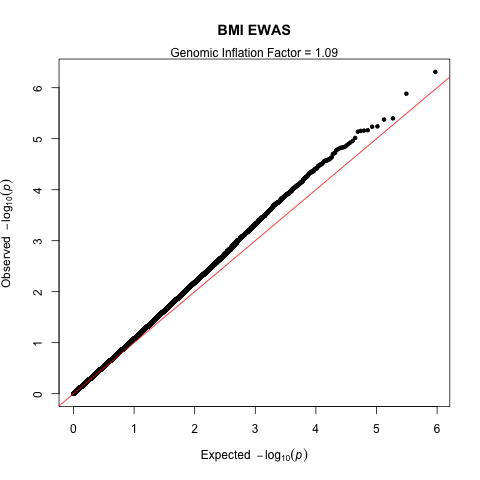

Supplement: Additional file 3: Figure S3. — Q-Q plots of the EWAS results with (A) BMI (genomic inflation factor = 1.09), and (B) T2D (genomic inflation factor = 1.10). The red line shows the expected p values. 49.7 KB [file 13148_2016_177_MOESM3_ESM.zip › SupFig3a.png]

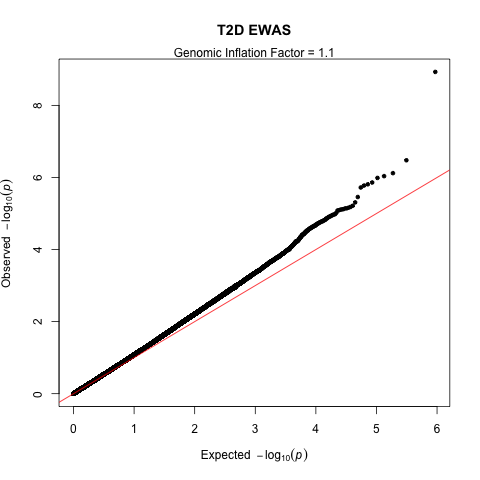

Supplement: Additional file 3: Figure S3. — Q-Q plots of the EWAS results with (A) BMI (genomic inflation factor = 1.09), and (B) T2D (genomic inflation factor = 1.10). The red line shows the expected p values. 49.7 KB [file 13148_2016_177_MOESM3_ESM.zip › SupFig3b.png]

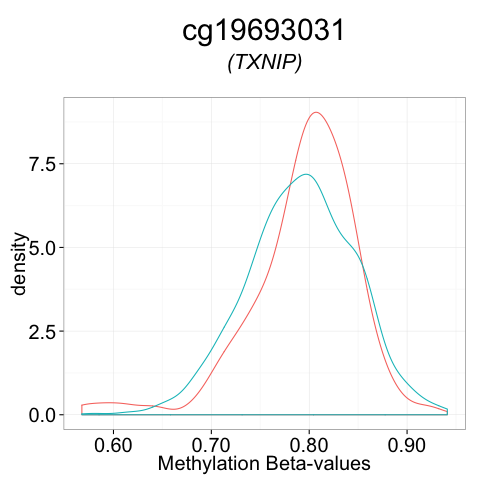

Supplement: Additional file 6: Figure S4. — Distribution of methylation beta value for the Qatari family study (red) and the TwinsUK cohort (blue). While the standardized BMI distribution was not different between the two samples (Kolmogorov-Smirnov p value>0.05), the distribution of six out of eight methylation values at the tested probes were different in either location or shape (Kolmogorov-Smirnov p value<0.05) 309 KB [file 13148_2016_177_MOESM6_ESM.zip › SupFig4_1.png]

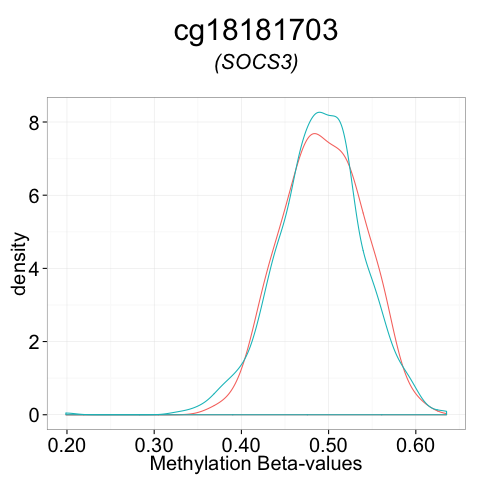

Supplement: Additional file 6: Figure S4. — Distribution of methylation beta value for the Qatari family study (red) and the TwinsUK cohort (blue). While the standardized BMI distribution was not different between the two samples (Kolmogorov-Smirnov p value>0.05), the distribution of six out of eight methylation values at the tested probes were different in either location or shape (Kolmogorov-Smirnov p value<0.05) 309 KB [file 13148_2016_177_MOESM6_ESM.zip › SupFig4_2.png]

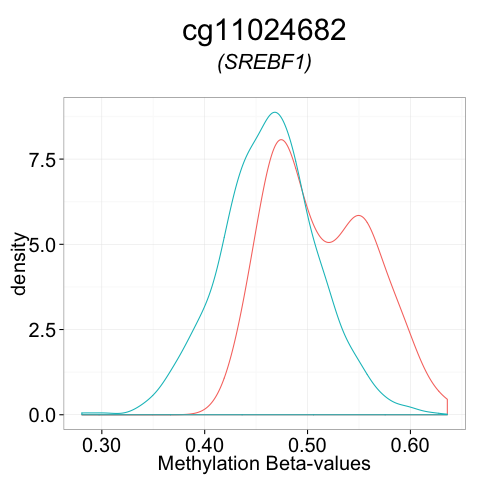

Supplement: Additional file 6: Figure S4. — Distribution of methylation beta value for the Qatari family study (red) and the TwinsUK cohort (blue). While the standardized BMI distribution was not different between the two samples (Kolmogorov-Smirnov p value>0.05), the distribution of six out of eight methylation values at the tested probes were different in either location or shape (Kolmogorov-Smirnov p value<0.05) 309 KB [file 13148_2016_177_MOESM6_ESM.zip › SupFig4_3.png]

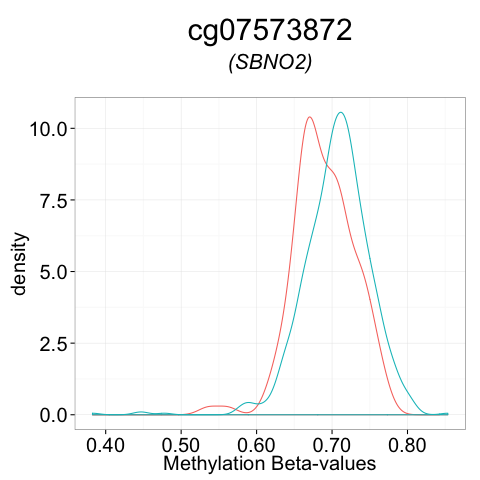

Supplement: Additional file 6: Figure S4. — Distribution of methylation beta value for the Qatari family study (red) and the TwinsUK cohort (blue). While the standardized BMI distribution was not different between the two samples (Kolmogorov-Smirnov p value>0.05), the distribution of six out of eight methylation values at the tested probes were different in either location or shape (Kolmogorov-Smirnov p value<0.05) 309 KB [file 13148_2016_177_MOESM6_ESM.zip › SupFig4_4.png]

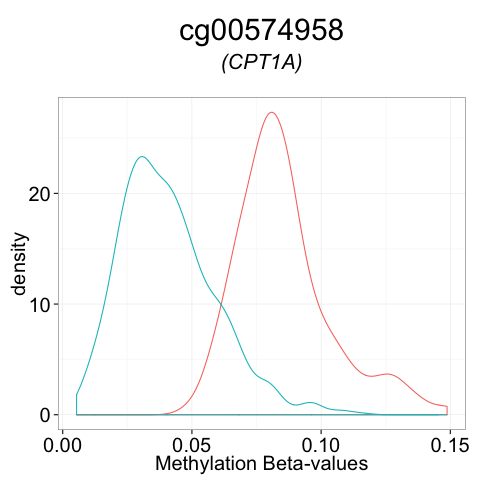

Supplement: Additional file 6: Figure S4. — Distribution of methylation beta value for the Qatari family study (red) and the TwinsUK cohort (blue). While the standardized BMI distribution was not different between the two samples (Kolmogorov-Smirnov p value>0.05), the distribution of six out of eight methylation values at the tested probes were different in either location or shape (Kolmogorov-Smirnov p value<0.05) 309 KB [file 13148_2016_177_MOESM6_ESM.zip › SupFig4_5.png]

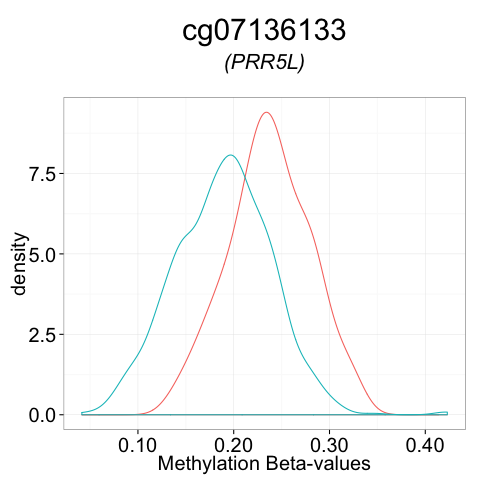

Supplement: Additional file 6: Figure S4. — Distribution of methylation beta value for the Qatari family study (red) and the TwinsUK cohort (blue). While the standardized BMI distribution was not different between the two samples (Kolmogorov-Smirnov p value>0.05), the distribution of six out of eight methylation values at the tested probes were different in either location or shape (Kolmogorov-Smirnov p value<0.05) 309 KB [file 13148_2016_177_MOESM6_ESM.zip › SupFig4_6.png]

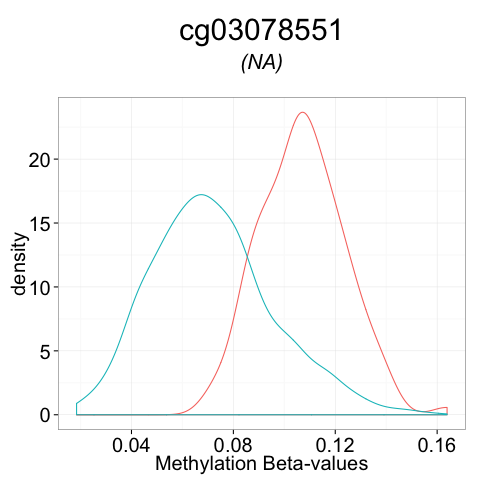

Supplement: Additional file 6: Figure S4. — Distribution of methylation beta value for the Qatari family study (red) and the TwinsUK cohort (blue). While the standardized BMI distribution was not different between the two samples (Kolmogorov-Smirnov p value>0.05), the distribution of six out of eight methylation values at the tested probes were different in either location or shape (Kolmogorov-Smirnov p value<0.05) 309 KB [file 13148_2016_177_MOESM6_ESM.zip › SupFig4_7.png]

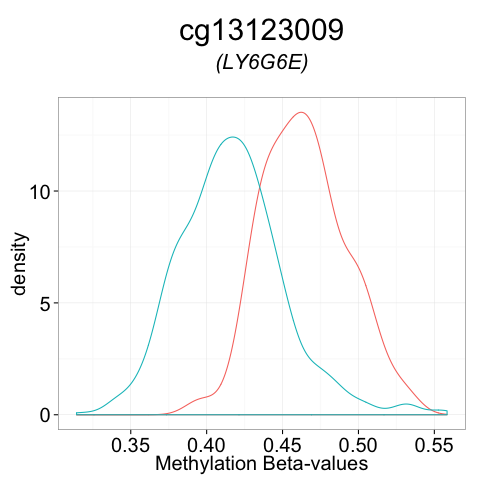

Supplement: Additional file 6: Figure S4. — Distribution of methylation beta value for the Qatari family study (red) and the TwinsUK cohort (blue). While the standardized BMI distribution was not different between the two samples (Kolmogorov-Smirnov p value>0.05), the distribution of six out of eight methylation values at the tested probes were different in either location or shape (Kolmogorov-Smirnov p value<0.05) 309 KB [file 13148_2016_177_MOESM6_ESM.zip › SupFig4_8.png]
